# High-resolution quantitative trait locus mapping for rice grain quality traits using genotyping by sequencing

**DOI:** 10.3389/fpls.2022.1050882

**Published:** 2023-01-12

**Authors:** Su-Kui Jin, Li-Na Xu, Qing-Qing Yang, Ming-Qiu Zhang, Shui-Lian Wang, Ruo-An Wang, Tao Tao, Lian-Min Hong, Qian-Qian Guo, Shu-Wen Jia, Tao Song, Yu-Jia Leng, Xiu-Ling Cai, Ji-Ping Gao

**Affiliations:** ^1^ JiangsuKey Laboratory of Crop Genomics and Molecular Breeding, College of Agriculture, Yangzhou University, Yangzhou, China; ^2^ Key Laboratory of Plant Functional Genomics of the Ministry of Education, College of Agriculture, Yangzhou University, Yangzhou, China; ^3^ Jiangsu Key Laboratory of Crop Genetics and Physiology, College of Agriculture, Yangzhou University, Yangzhou, China; ^4^ Jiangsu Co-Innovation Center for Modern Production Technology of Grain Crops, College of Agriculture, Yangzhou University, Yangzhou, China; ^5^ National Key Laboratory of Plant Molecular Genetics, Chinese Academy of Sciences (CAS) Center for Excellence in Molecular Plant Sciences, Shanghai Institute of Plant Physiology and Ecology, Chinese Academy of Sciences, Shanghai, China; ^6^ Shanghai Institutes for Biological Sciences, University of Chinese Academy of Sciences, Beijing, China; ^7^ Innovation Academy for Seed Design, Chinese Academy of Sciences, Beijing, China

**Keywords:** rice, grain quality, quantitative trait locus, genotyping by sequencing, high-density genetic map

## Abstract

Rice is a major food crop that sustains approximately half of the world population. Recent worldwide improvements in the standard of living have increased the demand for high-quality rice. Accurate identification of quantitative trait loci (QTLs) for rice grain quality traits will facilitate rice quality breeding and improvement. In the present study, we performed high-resolution QTL mapping for rice grain quality traits using a genotyping-by-sequencing approach. An F_2_ population derived from a cross between an elite *japonica* variety, Koshihikari, and an *indica* variety, Nona Bokra, was used to construct a high-density genetic map. A total of 3,830 single nucleotide polymorphism markers were mapped to 12 linkage groups spanning a total length of 2,456.4 cM, with an average genetic distance of 0.82 cM. Seven grain quality traits—the percentage of whole grain, percentage of head rice, percentage of area of head rice, transparency, percentage of chalky rice, percentage of chalkiness area, and degree of chalkiness—of the F_2_ population were investigated. In total, 15 QTLs with logarithm of the odds (LOD) scores >4 were identified, which mapped to chromosomes 6, 7, and 9. These loci include four QTLs for transparency, four for percentage of chalky rice, four for percentage of chalkiness area, and three for degree of chalkiness, accounting for 0.01%–61.64% of the total phenotypic variation. Of these QTLs, only one overlapped with previously reported QTLs, and the others were novel. By comparing the major QTL regions in the rice genome, several key candidate genes reported to play crucial roles in grain quality traits were identified. These findings will expedite the fine mapping of these QTLs and QTL pyramiding, which will facilitate the genetic improvement of rice grain quality.

## Introduction

Rice is one of the most important food crops that sustains approximately half of the world population. Recent global improvements in the standard of living have increased the demand for high-quality rice, straining the capacity of rice-producing areas worldwide (Cheng et al., 2007; Zhang, 2007; Fitzgerald et al., 2009). Thus, the improvement of rice quality is of high interest to plant biologists and plant breeders. Most grain-quality traits are complex quantitative traits controlled by multiple genes. With the development of molecular marker technology and construction of physical linkage maps in rice, it has become feasible to dissect complex polygenic traits in rice. Over the past two decades, considerable progress has been made in molecular marker and quantitative trait locus (QTL) analysis methods in rice, providing thousands of QTLs, some of which have been fine-mapped and cloned (Hao and Lin, 2010; Li et al., 2022). However, it remains difficult to identify mechanisms underlying rice grain quality traits, as most are polygenic and susceptible to environmental factors.

The grain quality traits in rice are determined by many variables, including milling traits, physical appearance, cooking and taste properties, and nutritional quality (Fitzgerald et al., 2009; Li et al., 2022). For example, the price of unbroken rice kernels (i.e., the milled whole kernel obtained from rough rice) is typically twice as that of broken grains (Childs, 2006). Therefore, milling quality is an economically important factor. Milling quality traits include brown rice recovery, milled rice recovery, whole grain recovery, head rice recovery, and the fraction of head rice (Septiningsih et al., 2003). Although several milling quality QTLs have been identified (Tan et al., 2001; Septiningsih et al., 2003; Aluko et al., 2004; Dong et al., 2004; Li et al., 2004; Jiang et al., 2005; Zheng et al., 2007; Lou et al., 2009; Nelson et al., 2011), no dominant, large-effect gene has been cloned and functionally characterized. Another critical grain appearance trait is chalkiness—opacity of rice endosperm—on the basis of which rice kernels are grouped into white core, white belly, and white back phenotypes. Chalkiness represents a primary index of grain quality because it affects not only grain appearance but also milling, taste, and nutritional quality (Fitzgerald et al., 2009; Zhao et al., 2022). After decades of effort, researchers have identified hundreds of QTLs for chalkiness distributed across the rice genome (He et al., 1999; Hao et al., 2009; Qin et al., 2009; Chen et al., 2016; Qiu et al., 2017; Zhu et al., 2018). According to the GRAMENE database, 82 QTLs for endosperm chalkiness have been identified, including 30 QTLs for percentage of white-core grains, 26 QTLs for degree of endosperm chalkiness, 12 QTLs for area of endosperm chalkiness, 11 QTLs for white-backed kernel, and 3 QTLs for basal white. Owing to the genetic complexity and instability of chalkiness, to date, only a few QTLs for chalkiness have been cloned. *Chalk5*, the first cloned QTL for chalkiness, located on chromosome 5, encodes a vacuolar H^+^-translocating pyrophosphatase (V-PPase) (Li et al., 2014). V-PPase activity is essential for establishing a proton gradient between the vacuolar lumen and cell cytosol. Elevated enzyme activity of Chalk5 protein disrupts the proton gradient homeostasis of the endomembrane trafficking system and alters the subcellular ultrastructure of the endosperm, forming air pockets and resulting in chalky grain (Li et al., 2014). Moreover, several QTLs related to chalkiness have been fine-mapped, such as *qPGWC-8*, *qACE-9*, and *qPCG1* (Guo et al., 2011; Gao et al., 2016; Zhu et al., 2018). These fine-mapped QTLs facilitate gene cloning and functional analysis. Apart from chalkiness, transparency is another index of grain appearance. There is less known about the genetic and physiological mechanisms of grain transparency, with only a few QTLs known to be associated with it, such as *qET-3*, *qET-6*, *qET-7*, *qET-8* (data from GRAMENE).

Starch is a high-molecular-weight glucose polymer composed of linear amylose and highly branched amylopectin. Starch, which constitutes 90% of the dry weight of milled rice, is a dominant factor that dramatically affects cooking and eating quality (Huang et al., 2021). Moreover, the milling quality and appearance quality of rice are also affected by starch. Granule-bound starch synthase (GBSSI; also called Waxy) is responsible for amylose synthesis. *Wx*, which encodes OsGBSSI, is a major QTL for amylose content, gel consistency, and pasting viscosity (Bao, 2014). High-amylose rice grains tend to be dry and hard after cooking, whereas low-amylose rice is soft and sticky after cooking. Mutations in the *Wx* gene reduce or eliminate amylose production, resulting in opaque white grains (Sano et al., 1985; Terada et al., 2002). Amylopectin biosynthesis is controlled synergistically by a combination of multiple isoforms of starch synthase (SS), starch-branching enzyme (SBE), and starch-debranching enzyme (DBE) (Tian et al., 2009; Huang et al., 2021). Among genes encoding amylopectin synthesis enzymes, *Soluble starch synthase IIa (SSIIa)* is a major QTL for gelatinization temperature (GT) and amylopectin structure (Gao et al., 2003; Nakamura et al., 2005; Gao et al., 2011). SSIIa is almost inactive in *japonica* varieties, with only 10% of the starch synthase activity in the soluble fraction of the developing endosperm, in comparison with *indica* varieties (Nakamura et al., 2005). When the *indica SSIIa* gene was introduced into *japonica* rice varieties, the transgenic plants showed increased GT and higher amylopectin side-chain length (Nakamura et al., 2005; Gao et al., 2011). Downregulation or loss of function of the genes encoding amylopectin synthesis enzymes typically result in obvious defects in starch synthesis, leading to abnormal endosperm phenotypes such as shrunken endosperm and chalkiness (Tanaka et al., 2004; Fujita et al., 2007; Zhang et al., 2011; Crofts et al., 2017; Lee et al., 2017).

Conventional breeding requires considerable time for selecting desirable phenotypes *via* hybridization over many generations. Marker-assisted selection (MAS) has greatly increased the speed and accuracy of phenotype selection in the breeding population (Tester and Langridge, 2010). Many of the previous genotyping strategies for QTL mapping and MAS used restriction fragment length polymorphism (RFLP) (Bernatzky and Tanksley, 1986), simple sequence repeat (SSR) markers (Litt and Luty, 1989), and random amplification of polymorphic DNA (RAPD) (Williams et al., 1990), among other techniques (Konieczny and Ausubel, 1993; Paran and Michelmore, 1993; Salimath et al., 1995; Vos et al., 1995; Desmarais et al., 1998). However, the development of next-generation sequencing technologies has made the whole-genome sequencing (WGS) of mapping populations feasible. Reduced-representation genome sequencing (RRGS), including restriction-site-associated DNA sequencing (RAD-seq) and genotyping by sequencing (GBS), generates a large number of single nucleotide polymorphisms (SNPs) for genetic analyses and genotyping (Baird et al., 2008; Beissinger et al., 2013). Compared with WGS, RRGS is promising for mapping populations because of its cost efficiency and abundant genotyping data output. GBS reduces genome complexity by using restriction enzymes to cleave the DNA into smaller fragments, which are coupled with barcoded adapters for sequencing. It is also useful when working with species with complex genomes, and it can accommodate large, polyploid genomes and species without a reference genome (Qi et al., 2018). GBS can offer high SNP coverage in gene-rich genomic regions in a highly cost-effective manner by using appropriate restriction enzymes (Chung et al., 2017). With GBS approaches, the rapid discovery of sequence-based molecular markers has been successful in constructing high-density genetic maps in maize, oat, chickpea, and ramie (Chen et al., 2014; Huang et al., 2014; Jaganathan et al., 2015; Liu et al., 2017). Consequently, GBS is becoming more common in genetic mapping studies, genome-wide association studies, genomic selection, polyploidy studies, and genetic diversity and phylogeny studies (He et al., 2014; Limborg et al., 2016; Chung et al., 2017; Kumar et al., 2021).

The GBS technique has been widely used to identify a large number of SNPs, construct high-density linkage maps, examine genetic diversity, and map QTLs for various agronomic traits in rice. For instance, two populations of interspecific introgression lines derived from a cross between a tropical *japonica* upland cultivar and two different wild rice varieties were developed and genotyped using GBS and SSR markers (Arbelaez et al., 2015). A breeding population comprising 369 elite tropical rice breeding lines were genotyped with 71,710 SNPs using GBS, and 52 QTLs for 11 yield-related agronomic traits were identified through genome-wide association mapping studies (Begum et al., 2015). Using a high-density GBS-based SNP linkage map, a total of 85 QTLs for nine traits related to salinity tolerance were identified from a population of 187 recombinant inbred lines (RILs) (De Leon et al., 2016). Rice landraces are important genetics resources for rice breeding. GBS was performed to analyze the genetic diversity and population structure of 96 rice landraces from Kerala, India, revealing significant genetic differentiation (Vasumathy et al., 2020; Peringottillam et al., 2022). However, few studies have reported using GBS to identify QTLs related to rice grain quality. A previous study dissected the genetic basis of rice grain quality developed an RIL population from an inter-subspecific cross between *indica* rice PYZX and *japonica* rice P02428; a high-density genetic map was constructed using 2,711 recombination bin markers generated from GBS, and 12 QTL clusters for grain shape and chalkiness were identified, including one for chalkiness and one for both chalkiness and grain shape (Chen et al., 2016). Recently, a study reported that, using GBS, 14 QTLs for grain protein content were identified with an RIL population derived from a cross between Huanghuazhan and Jizi1560, and one stable QTL—*qGPC1-1*—was delimited to a ~862 kb interval (Wu et al., 2020).

In the present study, we employed a genotype-by-sequencing technique on a *japonica × indica* F_2_ population to yield a high-density genetic map ideal for mapping rice grain quality traits. The *japonica* rice Koshihikari cultivar is a premium short-grain rice cultivar developed in 1956. Since 1979, Koshihikari has been the most widely grown cultivar in Japan, preferred commercially for its sticky and chewy texture (Kobayashi et al., 2018). Nona Bokra, an *indica* variety originating from India, is a well-known salt-tolerant donor in rice breeding (Ren et al., 2005; Takai et al., 2007), but its grain quality is generally considered poor. In this study, we generated a genetic map for an F_2_ population derived from a cross between the Koshihikari and Nona Bokra cultivars and conducted QTL mapping for seven grain quality traits. The study aimed to precisely identify the genomic regions underlying crucial rice quality traits to facilitate future candidate gene identification and genetically informed breeding programs.

## Materials and methods

### Mapping population and phenotypic data

The parental line Koshihikari is an elite *japonica* rice cultivar, and the other parental line, Nona Bokra, is an *indica* variety with poor grain quality. An F_2_ population consisting of 529 plants was developed from a cross between Koshihikari and Nona Bokra. F_2_ seeds were germinated and planted in Shanghai, China (121°24′E, 31°00′N) in the summer of 2017. The F_2_ plants were grown under typical field conditions for rice production. A total of 384 individuals from the F_2_ population was used to construct the linkage map, of which 172 were used for phenotyping. The grain quality traits were measured from ~200 fully filled grains per individual. The quality traits of milled rice grains were analyzed using the SC-E rice apparent quality analysis system (Hangzhou WSeen Detection Technology Co., Ltd., China) following the manufacturer’s instructions. Seven grain quality traits were analyzed in this study: percentage of whole grain (WG; the percentage of whole grains in a milled rice sample), percentage of head rice (HR; the percentage of grains that are 75% or more of the average length of whole grains in a milled rice sample), percentage of area of head rice (AHR; the percentage of the area of grains that are 75% or more of the average whole-grain length to the area of the milled rice sample), transparency (TP; in the SC-E rice apparent quality analysis system, the transparency of the head rice is classified into five grades: 1 to 5 corresponding to >0.70, 0.70–0.60, 0.60–0.45, 0.45–0.30, and <0.30 of light transmittance, respectively), percentage of chalky rice (CR; the percentage of head rice grains with an opaque, chalky appearance in a sample), percentage of chalkiness area (CA; the average value in a sample of the percentage of the area of chalkiness in a head rice grain to the area of the head rice grain), and the degree of chalkiness (DC; the percentage of the total chalky area of chalky rice grains to the total area of head rice grains in a sample). Frequency distribution graphs for each trait were constructed in Microsoft Excel 2010. Pearson correlation coefficients among the WG, HR, AHR, TP, CR, CA, and DC values were calculated using the CORREL function in IBM SPSS Statistics (Version 25).

### Genotyping-by-sequencing library preparation

Young leaf samples were collected from 384 F_2_ individuals for DNA extraction. All the samples were immediately frozen in liquid nitrogen and preserved at -80°C until extraction. The total genomic DNA was extracted using a DNA extraction kit (DNeasy 96 Plant Kit, QIAGEN) according to the manufacturer instructions. DNA concentration was measured using a Qubit fluorometer (INVITROGEN). The GBS libraries were constructed as described in Qi et al. (2018) using restriction enzymes PstI and MspI. The concentration of each sample was adjusted to 8–15 ng/µL. In total, 11,520 ng of the DNA from 384 samples was pooled and sequenced on the Illumina platform. A total of 886,768,436 paired-end raw reads and 800,160,486 paired-end clean reads were obtained.

### Genetic linkage map construction

GBS read processing and SNP calling were performed as described in Qi et al. (2018). Reads from each sample were aligned to *Oryza sativa* MSU7.0 (downloaded from Phytozome) using Bowtie2 (–maxins 500 –no-discordant –no-mixed), and SNP calling was performed using the Unified Genotyper (-dcov 1000) function within the Genome Analysis Toolkit (GATK) (DePristo et al., 2011). SNP filtering included the removal of SNPs with three or more alleles (Qi et al., 2018) and a quality-by-depth (QD) score >10. SNPs with a read depth of at least 8× were converted to the mapping scores A, B, H, D (A or H) and C (B or H) (Qi et al., 2018). SNPs within the same GBS tag were consolidated to a single marker. Markers with more than 30% of missing data were removed.

Chi-square tests were used to test the deviation of the segregation ratio of each SNP from the expected 1:2:1 ratio across the progenies. The markers with a *p*-value ≥ 1e-5 were chosen for map construction. The genetic map construction using MSTMap and MapMaker was performed using the approach described by Qi et al. (2018).

### Quantitative trait locus analysis

QTL mapping was done using WinQTLCart 2.5. The population type for HH maps was set as “SF2.” Composite interval mapping with a walking speed of 0.5 cM was used to identify QTLs. The logarithm of the odds (LOD) threshold for each trait for significant QTLs *(p* ≤ 0.05) was determined based on 1000 permutations.

### Development of chromosome segment substitution lines

To validate the genetic effects of QTLs identified in this study, a population of chromosome segment substitution lines (CSSLs) derived from the recurrent parent Koshihikari and the donor parent Nona Bokra was developed. Koshihikari was crossed with Nona Bokra, and the resulting F_1_ plants were backcrossed to Koshihikari three times to generate BC_3_F_1_ plants. We performed foreground selection from F_1_ to the BC_2_F_1_ generation to select heterozygous target chromosome segments using 127 simple sequence repeat markers, but not background selection. Both foreground and background selections were performed in BC_3_F_1_, BC_3_F_2_, and BC_3_F_3_ generations, and 147 homozygous CSSLs were identified in the BC_3_F_3_ population. Nine homozygous CSSLs were selected to confirm the genetic effect of QTLs on chromosome 6, 7, and 9, respectively.

## Results

### Sequencing of the F_2_ population

**Figure 1:**
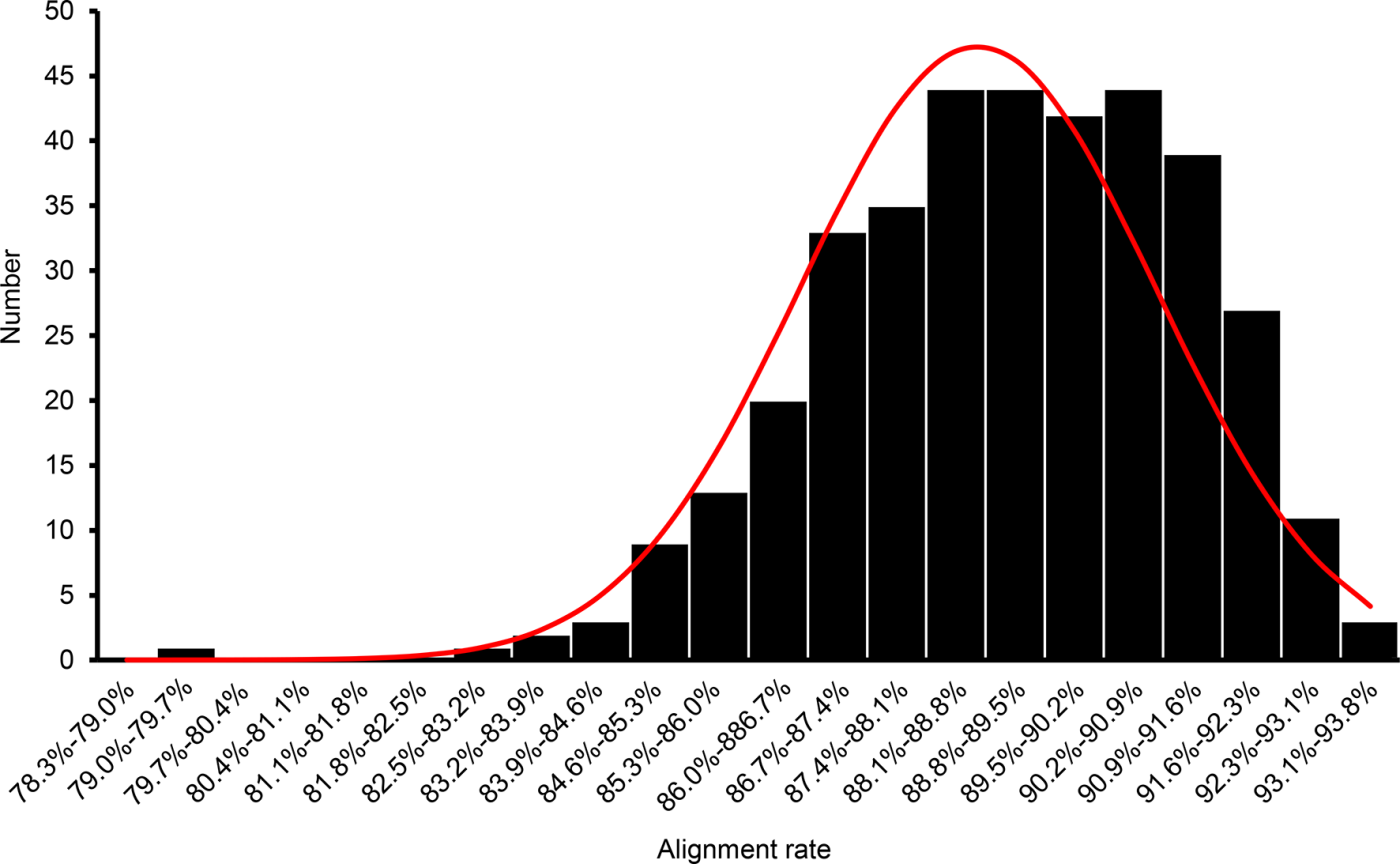
Frequency distribution of the percentage of raw genotyping-by-sequencing reads of the F_2_ population mapped to the reference genome.

To identify the QTLs for grain quality traits, an F_2_ population derived from a cross between an elite *japonica* variety, Koshihikari, and an *indica* variety, Nona Bokra. In total, 384 out of 529 F_2_ plants were randomly selected to construct a GBS library. The GBS library was sequenced by Illumina HiSeq X10 platform and generated a total of 886,768,436 reads (248 GB sequence data). Samples with less than 400,000 reads were excluded from analysis. The average and median number of reads per sample of the remaining 371 samples was 2,164,244 and 2,177,405, respectively (**Supplemental Table 1**). The trimmed reads were aligned against the *Oryza sativa* MSU7.0 (downloaded from Phytozome) reference genome sequence, and the average number of mapped reads per individual in the F_2_ population was 1,932,680 (89.30% of the total number of reads; **Figure 1**, **Supplemental Table 1**).

### Construction of a high-density genetic map

**Figure 2:**
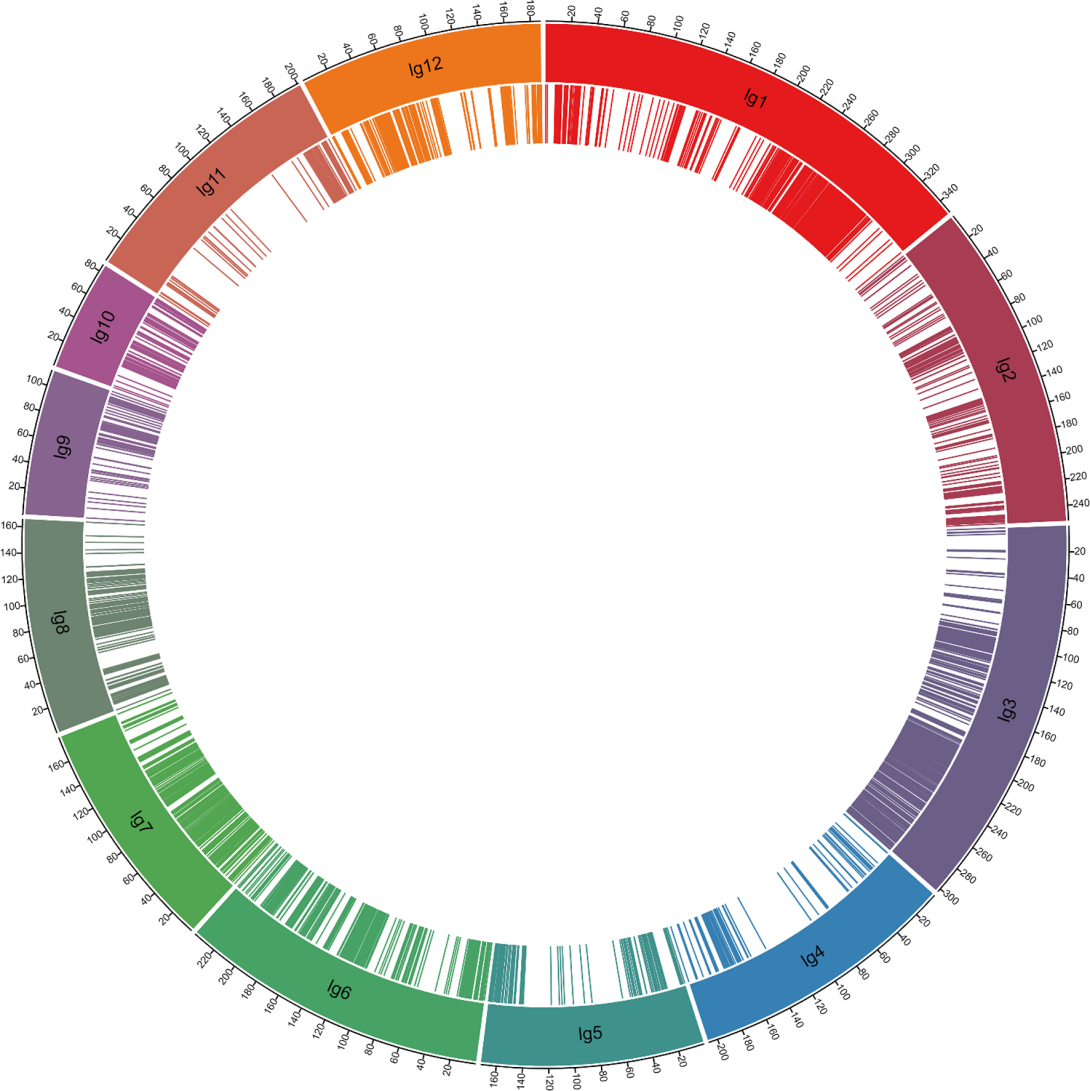
High-density genetic map constructed using the genotyping-by-sequencing approach. The genetic distances are expressed in centimorgans (cM). The bin markers’ position is indicated as bars in the inner ring.

A total of 3,830 SNP markers formed 12 linkage groups, with a total length of 2,456.4 cM and 2,984 SNP markers as bin markers, mapped to the 12 rice chromosomes (**Supplemental Table 2**, **Figure 2**). To conduct genetic analysis, the recombination maps were divided into a bin map, and all chromosomes of the 371 F_2_ individual populations were aligned and compared over intervals of a minimum of 100 kb. Adjacent 100 kb intervals with the same genotype across the entire F_2_ population were considered as a single recombination bin. The SNP markers were randomly distributed in the genetic map, with 102–569 binned SNP markers per linkage group. The individual linkage groups ranged from 85.7 to 351.3 cM in length, with an average genetic distance of 0.82 cM and a maximum distance of 46.5 cM (**Supplemental Table 2**). A total of 26 intervals had genetic distances greater than 10 cM (**Supplemental Table 2**).

### Phenotypic characterization of grain quality

The F_2_ population developed from the cross between Koshihikari and Nona Bokra cultivars was used for phenotypic measurements. Because inter-subspecies hybridization between *japonica* and *indica* rice varieties often leads to low levels of fertility or even sterility, only 172 out of the 384 F_2_ plants used to construct the GBS library produced sufficient seeds for phenotyping. Phenotyping data including WG, HR, AHR, TP, CR, CA, and DC were collected from 172 individuals (**Supplemental Table 3**).

**Figure 3:**
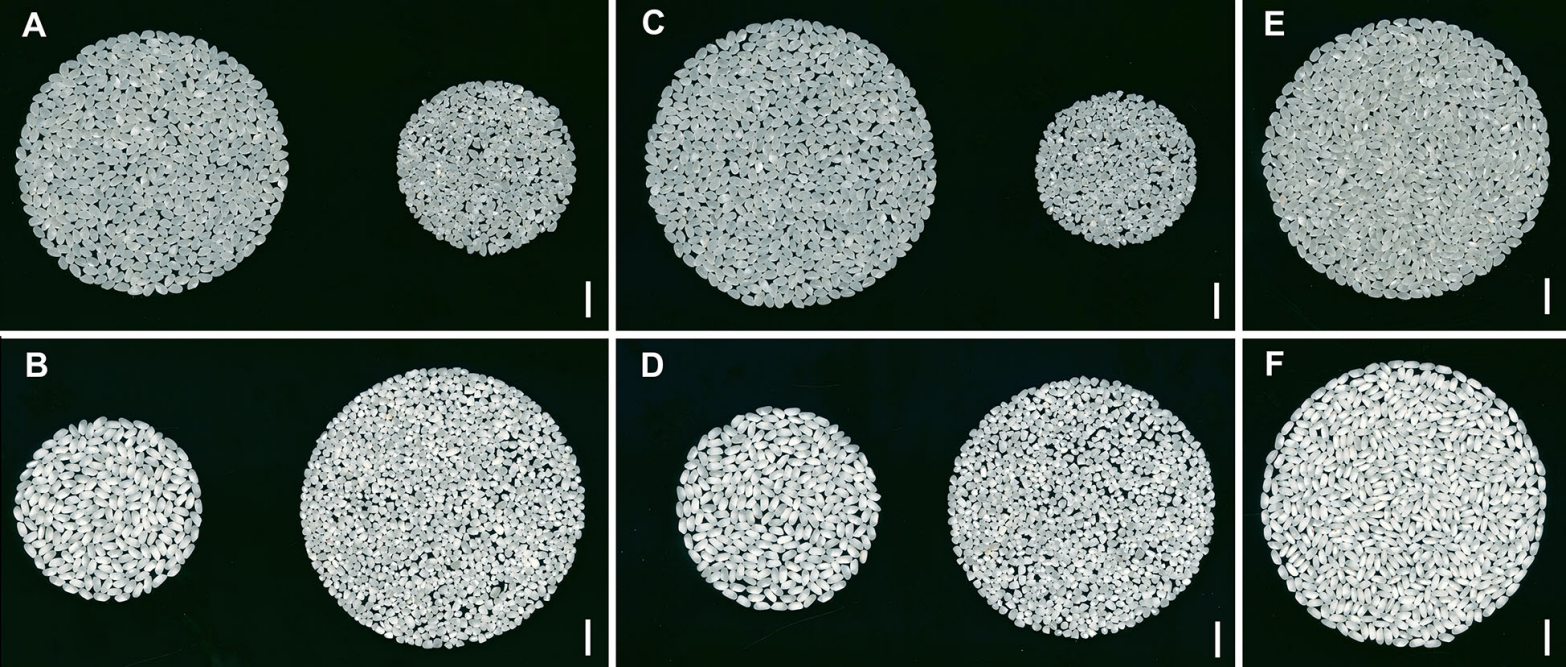
Phenotypes of milled rice from Koshihikari and Nona Bokra. **(A, B)** The whole grain appearance of Koshihikari **(A)** and Nona Bokra **(B)**. Whole grains are on the left, and broken grains are on the right. **(C, D)** The head rice appearance of Koshihikari **(C)** and Nona Bokra **(D)**. Head rice grains are on the left, and non-head rice grains are on the right. **(E, F)** The difference in chalkiness between head rice from Koshihikari **(E)** and Nona Bokra **(F)**. Scale bar: 1 cm.

There were significant differences between rice grain quality traits between the two parent varieties (**Figure 3**, **Table 1**). Koshihikari exhibited significantly higher WG, HR, AHR than Nona Bokra, whereas Nona Bokra showed significantly higher TP, CR, CA, and DC than Koshihikari. Notably, the CR and CA values in Nona Bokra were greater than 97%. These results indicate that Koshihikari is significantly superior to Nona Bokra in both milling quality and appearance quality.

**Table 1:** Description of the grain quality traits of the two parental lines and the F_2_ population.

Traits	Parental line (Mean ± SD)			F_2_ population				
	Koshihikari	Nona Bokra	*p* value	Range	Mean ± SD	Coefficient of variation	Skewness	Kurtosis
WG (%)	82.04 ± 9.16	32.48 ± 2.70	0.0008	14.22−98.85	83.31 ± 13.08	0.16	-1.74	4.16
HR (%)	86.35 ± 8.37	34.84 ± 3.24	0.0006	15.95−99.43	86.33 ± 12.79	0.15	-1.91	5.13
AHR (%)	93.44 ± 4.54	56.25 ± 3.67	0.0004	34.85−99.78	92.46 ± 8.55	0.09	-2.77	12.09
TP	2.33 ± 0.58	5.00 ± 0.00	0.0013	2.00−5.00	4.30 ± 0.84	0.20	-0.68	-1.06
CR (%)	26.77 ± 3.83	97.67 ± 1.09	<0.0001	21.54−100.00	89.03 ± 18.14	0.20	-1.99	3.28
CA (%)	25.53 ± 3.85	97.83 ± 1.00	<0.0001	21.6−100.00	89.05 ± 18.18	0.20	-1.98	3.24
DC (%)	8.47 ± 1.31	51.93 ± 3.09	<0.0001	5.85− 83.62	52.95 ± 21.21	0.40	-0.59	-0.80

SD, standard deviation; WG, percentage of whole grain; HR, percentage of head rice; AHR, percentage of area of head rice; TP, transparency; CR, percentage of chalky rice; CA, percentage of chalkiness area; DC, degree of chalkiness.

**Figure 4:**
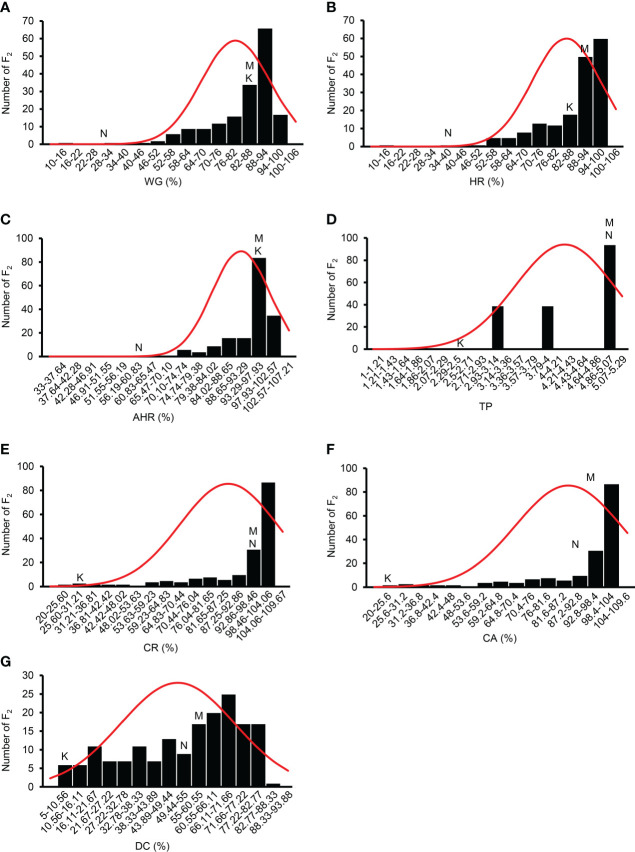
Frequency distribution of the grain quality traits of the F_2_ population. **(A)** WG, percentage of whole grain; **(B)** HR, percentage of head rice; **(C)** AHR, percentage of the area of head rice; **(D)** TP, transparency; **(E)** CR, percentage of chalky rice; **(F)** CA, percentage of chalkiness area; **(G)** DC, degree of chalkiness; K, Koshihikari; N, Nona Bokra; M, median.

Among the F_2_ population, the WG, HR, AHR, TP, CR, CA, and DC of 172 individuals showed significant phenotypic differences, indicating a wide range of variation (**Figure 4**, **Table 1**). As indicated in the frequency distribution graphs (**Figure 4**) and the range of values for each trait in the F_2_ population (**Supplemental Table 3**, **Table 1**), the median values of the traits HR, CA, and DC in the F_2_ population were outside the range observed in the parent varieties. Among the F_2_ population, most individuals were superior to the parent varieties in terms of WG, HR, and AHR and inferior to the parent varieties regarding TP, CR, CA, and DC (**Supplemental Table 3**, **Figure 4**). The grain quality traits showed negative skewness, indicating that the population distribution was negatively skewed (**Table 1**). The grain quality traits, except TP and DC, showed positive kurtosis. Of the seven grain quality traits AHR showed the highest kurtosis, indicating that the population distribution for AHR had a sharper peak than that of other traits (**Table 1**).

### Correlation of rice quality traits

The rice grain quality trait values varied widely within the F_2_ population (**Supplemental Table 3**). Correlations among all traits (**Table 2**) revealed that WG was highly positively correlated with HR and AHR. CR was highly positively correlated with CA and DC. In contrast, TP was negatively correlated with DC. The correlation among rice grain quality traits suggests the complex genetic regulation of these traits.

**Table 2:** Pearson correlation matrix of rice quality traits measured in a F_2_ population derived from the cross between Koshihikari and Nona Bokra rice varieties.

	WG	HR	AHR	TP	CR	CA	DC
WG	1						
HR	0.9898**	1					
AHR	0.9747**	0.9889**	1				
TP	0.1025	0.1223	0.1267	1			
CR	-0.0868	-0.0981	-0.0900	-0.3179**	1		
CA	-0.0840	-0.0959	-0.0880	-0.3180**	0.1000**	1	
DC	-0.1125	-0.1182	-0.1122	-0.6577**	0.8742**	0.8734**	1

WG, percentage of whole grain; HR, percentage of head rice; AHR, percentage of area of head rice; TP, transparency; CR, percentage of chalky rice; CA, percentage of chalkiness area; DC, degree of chalkiness. ^**^ indicates statistical significance at the level of 1%.

### Quantitative trait locus mapping of rice quality traits

**Figure 5:**
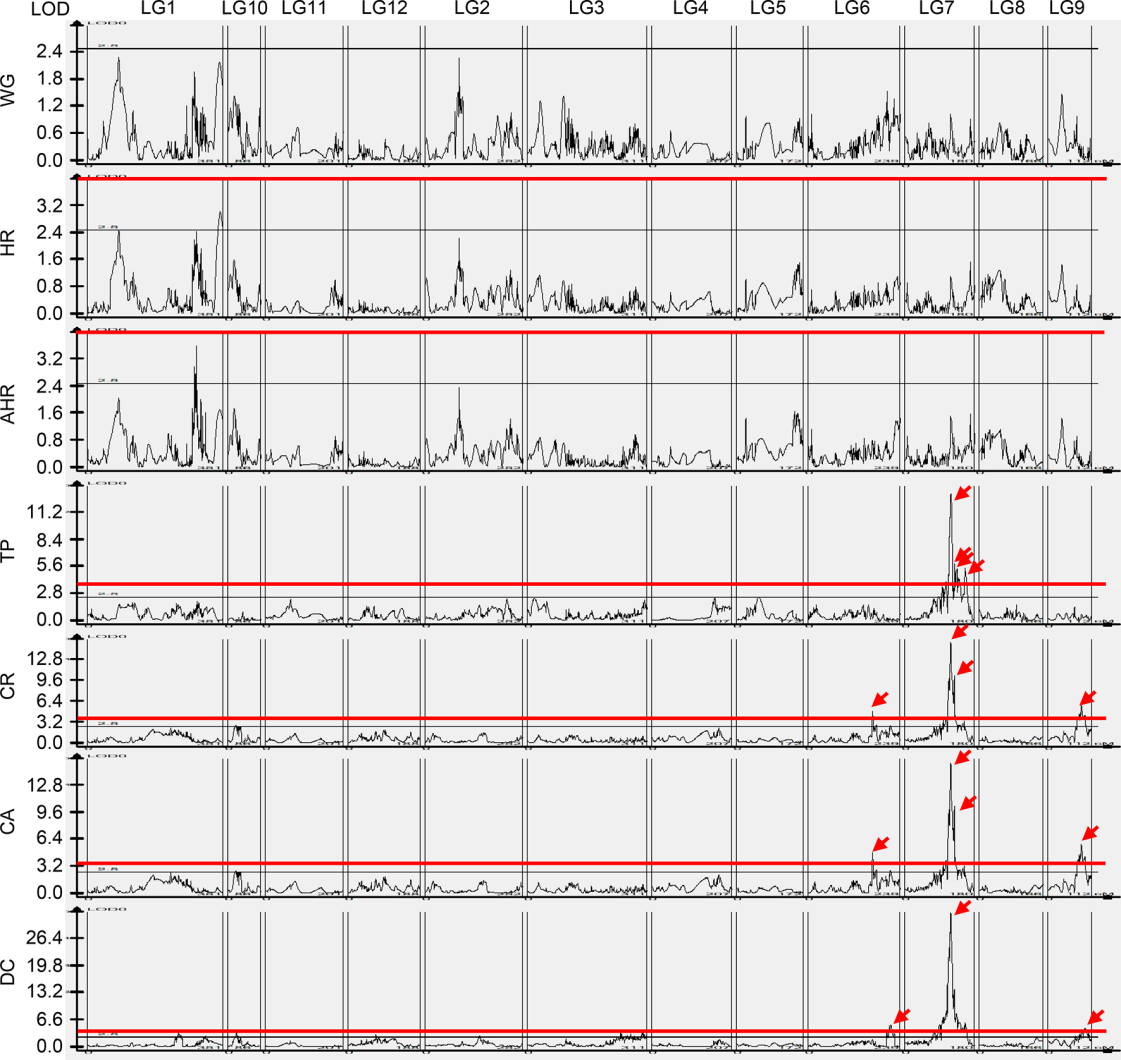
Quantitative trait loci for rice grain quality traits. The black line indicates LOD = 2.5, the red line indicates LOD = 4, and the red arrow indicates the LOD peaks of QTLs >4. WG, percentage of whole grain; HR, percentage of head rice; AHR, percentage of the area of head rice; TP, transparency; CR, percentage of chalky rice; CA, percentage of chalkiness area; DC, degree of chalkiness.

We identified 60 QTLs for the rice grain quality traits, except WG (**Figure 5**). No QTL was identified for WG, and only one QTL was detected for HR and AHR, respectively. Furthermore, by applying a 99% confidence interval (CI) for statistical significance, we focused on QTLs with LOD scores >4. The LOD values for the HR and AHR QTLs were less than 4; therefore, we conducted no further analysis on these traits. We did find QTLs with LOD >4 for TP, CR, CA, and DC, with LOD values ranging from 4.07 to 32.70 (**Table 3**, **Figure 5**). On the basis of these LOD values, we identified four QTLs for TP, four QTLs for CR, four QTLs for CA, and three QTLs for DC.

**Table 3:** QTLs identified for rice quality traits with LOD scores >4 in an F_2_ population derived from a cross between Koshihikari and Nona Bokra rice varieties.

Traits	QTL	Chr.	LOD Value	Left Marker	Peak Marker	Right Marker	QTL interval size (bp)	99% CI (cM)	Additive effect	Var.%	NO. of genes in QTL interval
Transparency	*TP-1*	7	4.07	15050264	14537854	8878341	6171905	97.6~108.3	-0.7217	0.18	341
	*TP-2*	7	13.17	6641297	6037460	5978310	662987	115.3~119.9	0.6635	45.79	33
	*TP-3*	7	5.9	5549865	5150303	4981427	568438	126.6~129.1	-0.7903	0.01	69
	*TP-4*	7	5.46	3439599	3336963	2516201	923398	151.9~163.9	-0.2957	7.7	59
Percentage of chalky rice	*CR-1*	6	4.86	22401613	22843130	23094605	692992	164.3~167.7	8.7227	5.71	40
	*CR-2*	7	15.54	6641297	6110977	6037460	603837	116.3~118.7	-9.552	35.71	26
	*CR-3*	7	10.3	5549865	–	5150303	399562	124.6~127.3	-8.1054	25.48	53
	*CR-4*	9	4.57	18050265	19075351	21382492	3332227	73.8~96.8	-1.9805	7.88	412
Percentage of chalkiness area	*CA-1*	6	4.82	22401613	22843130	23070149	668536	164.3~167.2	8.6934	5.75	39
	*CA-2*	7	15.5	6641297	6110977	6037460	603837	115.8~118.7	-9.5921	35.68	26
	*CA-3*	7	10.32	5389107	–	5150303	238804	124.3~127.3	-8.1512	25.54	28
	*CA-4*	9	4.59	18050265	19075351	21382492	3332227	73.8~96.3	-2.0465	7.95	412
Degree of chalkiness	*DC-1*	6	5.45	26660749	27507139	28002526	1341777	207.0~219.4	9.4554	7.75	120
	*DC-2*	7	32.7	6228705	6110977	6037460	191245	117.0~118.7	-14.1332	61.64	10
	*DC-3*	9	4.27	20330550	–	20726639	396089	84.8~89.9	-0.664	5.45	44

Chr, chromosome; CI, confidence intervals; Var, variance.

Among the four QTLs for TP, *TP-2* showed positive additive effects, whereas the *TP-1*, *TP-3*, and *TP-4* showed negative additive effects (**Table 3**). *TP-2* increased the transparency values conferred by alleles from Koshihikari, whereas *TP-1*, *TP-3*, and *TP-4* decreased the transparency values conferred by alleles from Koshihikari. The phenotypic variation explained (PVE) of the QTLs for TP ranged from 0.01% to 45.79%. The three QTLs for CR had negative additive effects, indicating that the majority, except *CR-1*, decreased the percentage of chalky rice conferred by alleles from Koshihikari. The PVE of the QTLs for CR ranged from 5.71% to 35.71%. For CA, the QTLs *CA-2*, *CA-3*, and *CA-4* decreased the percentage of chalkiness area conferred by alleles from Koshihikari, and *CA-1* increased the percentage of chalkiness area conferred by alleles from Koshihikari. The PVE of the QTLs for CA ranged from 5.75% to 35.68%. Three QTLs were identified for DC, of which *DC-2* and *DC-3* decreased the degree of chalkiness conferred by alleles from Koshihikari, and *DC-1* increased the degree of chalkiness conferred by alleles from Koshihikari. The PVE of the QTLs for DC ranged from 5.45% to 61.64% (**Table 3**).

**Figure 6:**
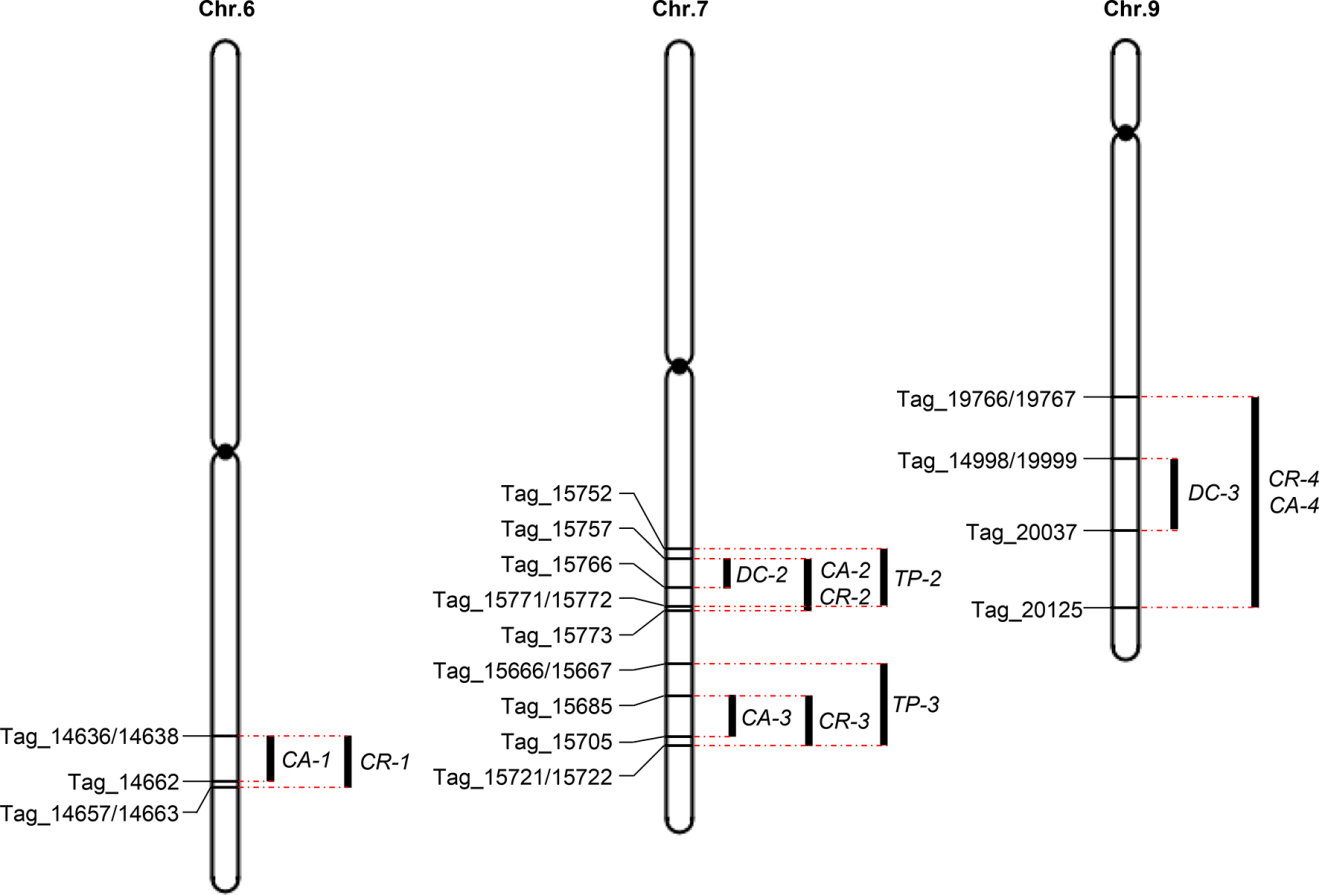
QTL clusters for rice grain quality traits. Black bars indicate QTLs identified using the composite interval mapping function in QTL Cartographer. Markers on the boundaries of QTLs are identified. Red dashed lines indicate the boundaries of overlapping QTLs.

The intervals of a number of these QTLs overlapped (**Figure 6**), indicating significant correlations across the four traits. We found that *CR-4* and *CA-4* had a shared interval on chromosome 9. Notably, the interval of *DC-3* was contained within the interval of *CR-4* and *CA-4*. *CR-2* and *CA-2* had a shared interval on chromosome 7. The interval of *DC-2* was contained within the interval of *CR-2* and *CA-2*, which was in turn contained within the interval of *TP-2*. Moreover, the interval of *CA-1* was contained within the interval of *CR-1*. The interval of *CA-3* was contained within the interval of *CR-3*, which was contained within the interval of *TP-3*. These results suggest that the QTLs that shared the same interval are likely regulated by a single gene. Moreover, of the 15 QTLs, only one QTL, *TP-1*—located on chromosome 7—overlapped with the previously reported QTLs *qDEC7* and *qPGWC7* (Zhou et al., 2009; Chen et al., 2016).

**Figure 7:**
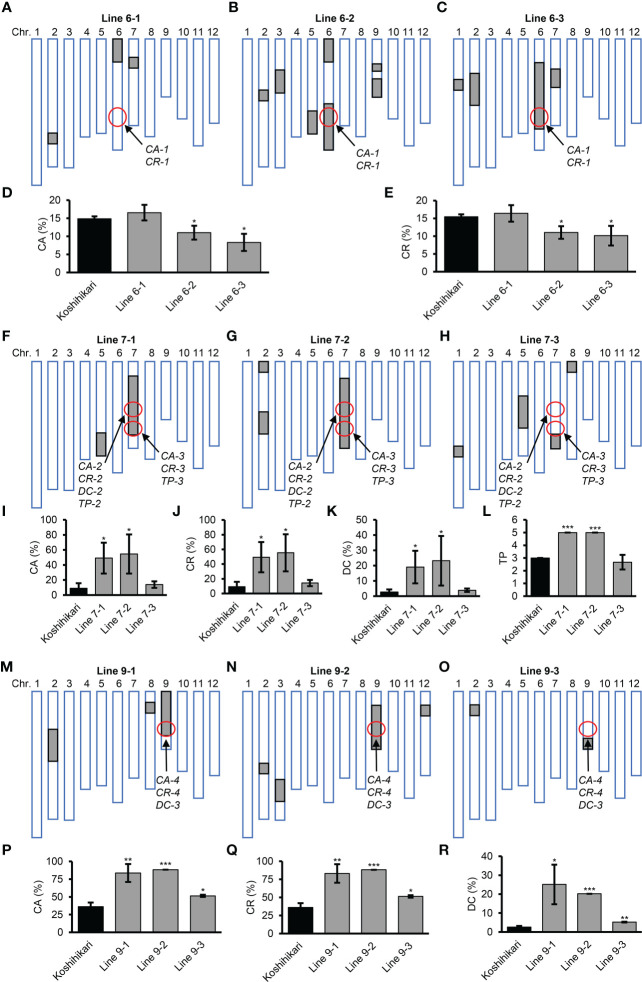
Grain quality traits of chromosome segment substitution lines. **(A−C, F−H, M−O)** Diagrams showing the genotypes of nine CSSLs. White boxes represent homozygous segments from Koshihikari; gray boxes represent homozygous segments from Nona Bokra; the positions of the indicated QTLs are circled. **(D, E, I−L, P−R)** Grain quality traits of CSSLs. CA, percentage of chalkiness area; CR, percentage of chalky rice; DC, degree of chalkiness; TP, transparency. Results are mean ± SD of three biological replicates. *p <0.05, **p <0.01, ***p <0.001, Student’s *t*-test.

To validate the genetic effects of QTLs in the four QTL clusters, nine CSSLs were selected from a population of CSSLs derived from crossing the recurrent parent Koshihikari and the donor parent Nona Bokra (**Figure 7**). Line 6-1 harbored the *CA-1* and *CR-1* alleles from Koshihikari, whereas Line 6-2 and 6-3 harbored these alleles from Nona Bokra (**Figures 7A–C**). Koshihikari and Line 6-1 exhibited higher CA and CR than Lines 6-2 and 6-3, confirming that the *CA-1* and *CR-1* alleles from Koshihikari increased CA and CR, respectively (**Figures 7D, E**, **Table 3**). There are two QTL clusters on chromosome 7 (**Figure 6**), but we did not obtain CSSLs containing only one QTL cluster. Lines 7-1 and 7-2 harbored DNA fragments from Nona Bokra, including the two QTL clusters on chromosome 7, while Line 7-3 harbored the DNA fragment from Koshihikari at the corresponding position (**Figures 7F–H**). Koshihikari and Line 7-3 exhibited lower CA, CR, DC, and TP than Lines 7-1 and 7-2, confirming that the *CA-2* and *CA-3*, *CR-2* and *CR-3*, *DC-2*, and *TP-2* and *TP-3* alleles from Koshihikari decreased CA, CR, DC, and TP, respectively (**Figures 7I–L**, **Table 3**). Notably, although *TP-2* and *TP-3* alleles from Koshihikari exerted positive and negative additive effects, respectively, their total additive effects were negative. Lines 9-1 and 9-2 harbored the *CA-4*, *CR-4*, and *DC-3* alleles from Nona Bokra, while Line 9-3 harbored these alleles from Koshihikari (**Figures 7M–O**). Koshihikari and Line 9-3 exhibited lower CA, CR, and DC than Lines 9-1 and 9-2, confirming that the *CA-4*, *CR-4*, and *DC-3* alleles from Koshihikari decreased CA, CR, and DC, respectively (**Figures 7P–R**, **Table 3**).

### Identification of candidate genes within quantitative trait loci related to rice quality traits

In this study, the average interval size of QTLs was 1,549 kb, with the longest interval of 6,172 kb, and the shortest interval of 191 kb (**Table 3**). The QTL with the shortest interval was *DC-2*, which also had the highest PVE. We then searched through all genes within the 15 QTL regions to identify candidate genes associated with rice grain quality traits. We found that the genes were concentrated on chromosomes 6, 7, and 9, and most were located on chromosome 7. There was a total of 1,074 genes within 15 QTLs, of which 616 genes were annotated and 458 genes were identified as expressed proteins, hypothetical proteins, transposons, and retrotransposon proteins (**Supplemental Table 4**). Because the QTLs in each QTL cluster may be controlled by one gene, we selected *CA-1*, *CA-3*, *DC-2*, and *DC-3* as well as *TP-1*, *TP-4*, and *DC-1* for further candidate gene analysis. Previous reports have shown that several kinds of genes are involved in the development of chalkiness and transparency in rice, including starch synthesis-related genes, storage protein biosynthetic pathway-related genes, and starch and storage protein biosynthesis-related transcription factor genes (Fan et al., 2022; Zhao et al., 2022). These were selected as candidate genes, and are listed in **Supplemental Table 5**.

## Discussion

### Genotyping by sequencing and linkage map

Linkage mapping is an important and powerful tool for the genetic analysis of quantitative traits. Identification of the genes associated with QTLs controlling a trait was challenging owing to the low resolution of early QTL maps with low-density markers. Since the first linkage map constructed using RFLP markers in rice (McCouch et al., 1988), several linkage maps have been produced using SSR (Golestan Hashemi et al., 2015; Bazrkar-Khatibani et al., 2019; Bhat et al., 2019; Jia et al., 2019) and SNP (Rahman et al., 2017; Jewel et al., 2019) markers in rice. However, these maps could not overcome the issues of either low-density linkage maps with unreasonable marker coverage or high-density linkage maps requiring considerably more time, manpower, and financial resources. In the present study, we generated a high-density linkage map using GBS technology and Illumina sequencing (**Figure 2**, **Supplemental Table 2**) and demonstrated the potential for further MAS. In this study, we identified QTLs for rice quality traits on the basis of extensive phenotyping of an F_2_ population derived from a cross between the Koshihikari and Nona Bokra varieties (**Table 3**, **Figure 5**). We used the GBS protocol as described by Qi et al. (2018) to genotype the mapping populations because of its potential for SNP discovery across the entire genome and the availability of a reference genome sequence (Nipponbare, MSU 7.0), which facilitated read alignment and SNP calling. GBS has been successfully applied in many different crops to obtain co-dominant SNP markers and to construct high-density genetic linkage maps with or without reference genomes (Gali et al., 2018; Qi et al., 2018; Qi et al., 2019). We constructed a high-density genetic linkage map with 3,830 markers in the F_2_ mapping population, of which 2,984 were used as the bin markers (**Supplemental Table 2**). In a similar study, a total of 9,303 SNP markers were identified and used to generate a high-density genetic linkage map by GBS in a rice population of 187 recombinant inbred lines developed from a cross between Bengal and Pokkali (De Leon et al., 2016). With larger populations, more recombination events will emerge, which requires much more marker coverage. However, when the population size is fixed, the markers tend to be saturated, and increasing the number of markers does not typically improve the accuracy of QTL mapping. The average distance between markers in our genetic linkage map was 0.82 cM, and SNP markers were distributed across the 12 chromosomes of rice (**Supplemental Table 2**). The high-density genetic linkage map will facilitate the fine-mapping of the identified QTLs for agronomic traits.

### Identification of quantitative trait loci for rice grain quality traits

In the present study, a total of 15 QTLs for rice grain quality with an LOD value greater than 4 were identified, including four QTLs for TP, four for CR, four for CA, and three for DC (**Table 3**). *CA-1*, *CR-1*, and *DC-1* were found to be located on chromosome 6; *CA-4*, *CR-4*, and *DC-3* were found to be located on chromosome 9, and the rest were found to be located on chromosome 7. A number of QTLs responsible for these traits have been previously identified. We found that at least seven QTLs are located in three intervals on chromosome 6: *qDEC-6a* and *qPGWC-6a* are located in the interval 10.7–13.5 cM (Li et al., 2003); *COAL10*, *qET-6*, *qPGWC-6b*, and *qSCE-6* are located in the interval 33–39.3 cM (Tan et al., 1999; Li et al., 2003); *qDEC-6b* is located in the interval 38.3–51 cM (Li et al., 2003). At least five QTLs are located in three intervals on chromosome 7: *qDEC7* and *qPGWC7* are located in the interval 19.2–20.8 cM (Qiu et al., 2017), *qDEC7* and *qPGWC7* are located in the interval 97.6–100.06 cM (Zhou et al., 2009; Chen et al., 2016), and *qET-7* is located in the interval 105.7–118.6 cM (Li et al., 2003). At least three QTLs are located in the same interval on chromosome 9: *qACE-9*, *qDEC-9*, and *qPGWC-9* are located in the interval 0–14 cM (Wan et al., 2005; Gao et al., 2016). Among the 15 QTLs identified in this study, only *TP-1*, which is located in the interval 97.6–108.3 cM on chromosome 7, overlapped with the previously reported QTLs *qDEC7* and *qPGWC7* (Zhou et al., 2009; Chen et al., 2016).

Identification of pleiotropic QTLs is especially useful for breeding, as multiple traits can be simultaneously selected for using a single genomic region. Of the 15 QTLs identified in this study, 12 QTLs formed four clusters (**Figure 6**, **Table 3**). The first cluster contained *CA-1* and *CR-1*; the second cluster comprised *DC-2*, *CA-2*, *CR-2*, and *TP-2*; the third cluster contained *CA-3*, *CR-3*, and *TP-3*; and the fourth cluster comprised *DC-3*, *CR-4*, and *CA-4*. This indicates that these grain quality traits are likely closely correlated. Therefore, these QTL clusters may be useful in improving rice quality traits by MAS.

### Accuracy of quantitative trait locus mapping

Because of the rapidly expanding population, increased grain yield is urgently needed in rice breeding. Grain weight, one of the rice grain yield components, is positively associated with grain size. Therefore, grain size is one of the most frequently studied traits in QTL mapping. In recent years, many QTLs for grain size have been identified (Li et al., 2018).

To verify the accuracy of our QTL mapping, we investigated QTLs associated with grain size in the F_2_ population (data not shown) to check whether any previously reported QTL were detected. We found that the intervals of several QTLs previously reported to be associated with grain size overlapped with the intervals of QTLs identified in the present study; for example, *qGW-4*, *qGW-5, qGW-8, qGW-9, qGW-10, qGW-13, qGW-14*, and *qGW-15* for grain width and *qGL-4* for grain length. *qGW-4* and *qGW-5* were found within the interval of *AQDH006* on chromosome 2 within a 63.5–105.1 cM region (data obtained from GRAMENE). *qGW-8*, *qGW-9*, and *qGW-10* were found within the interval of *qGL7* on chromosome 7 within a 97–117 cM region (Bai et al., 2010). *qGW-13*, *qGW-14*, and *qGW-15* were found within the interval of *GW8.1* on chromosome 8 within a 81–90 cM region (Xie et al., 2006). *qGL4* was found within the interval of our *qGL-4* on chromosome 4 within a 78–94 cM region (Kato et al., 2011). Moreover, the *PGL2* gene was found to be located within the interval of *qGW-4*. PGL2 is an atypical basic helix-loop-helix (bHLH) transcription factor that interacts with a typical bHLH protein APG, positively regulating grain length (Heang and Sassa, 2012). The *PGL2* gene was found to be located approximately 679 kb from the leftmost marker and 552 kb from the rightmost marker of *qGW-4*. These findings suggest that QTL mapping performed using a high-density genetic map in the present study is reliable and informative, with QTL peaks close to previously identified candidate genes.

### Importance of mapping rice quality traits

In recent years, the demand for high quality rice has become a leading issue with the improving standard of living worldwide. In this study, we focused on the milling quality and appearance quality. WG, HR, AHR, TP, CR, CA, and DC were measured in the F_2_ population derived from the cross between Koshihikari and Nona Bokra (**Supplemental Table 3**). As indicated in the frequency distribution graphs (**Figure 4**) and the range of F_2_ values for each trait (**Supplemental Table 3**), we found that the values of milling and appearance quality traits of the F_2_ population were more similar to Koshihikari milling quality and Nona Bokra appearance quality, respectively (**Figure 4**). Progenies with higher HR, CA, and DC than their parents were observed, indicating transgressive segregation for these traits. Such transgressive segregations are observed in many progenies derived from hybridization of genetically diverse parents because of allelic segregation and epistasis. In general, milling quality traits (WG, HR, and AHR) were highly correlated with each other, and appearance quality traits (CR, CA, and DC) were also highly correlated with each other (**Table 2**). These results indicate that grain quality traits show complex genetic regulation. To date, more than 82 QTLs for endosperm chalkiness have been reported. However, earlier sequencing strategies predicted that the intervals of most of these QTLs were more than 10 cM (data from GRAMENE). In this study, we used GBS technology to construct a high-density genetic map with an average marker interval of 0.82 cM and a maximum distance of 46.5 cM (**Supplemental Table 2**) with the goal of achieving more precise QTL mapping. In recent years, high-density linkage maps and QTL analysis has become incredibly useful for genetically informed breeding strategies. For example, a GBS-based high-density linkage map, QTL analysis, and associated candidate genes for stem bark traits in ramie has contributed to crop improvement (Liu et al., 2017). Highly significant and reproducible QTLs for several agronomic and seed quality traits of breeding importance in pea were also identified using a high-density linkage map (Gali et al., 2018). High-density linkage maps can be applied in mapping QTLs for traits related to growth, development, and flowering in switchgrass (Ali et al., 2019). Our explicit aim here is to harness these developing molecular tools to accelerate rice breeding for crucial and emerging traits.

### Identification of candidate genes

In this study, 15 QTLs associated with transparency, percentage of chalky rice, percentage of chalkiness area, and degree of chalkiness were found to be distributed on chromosome 6, 7, and 9 (**Table 3**). There were 616 annotated genes within these QTLs. Annotation of these genes showed that they are involved in cellular process, metabolic process, catalytic activity, and DNA binding (**Supplemental Table 4**). Starch synthesis-related genes, storage protein biosynthetic pathway-related genes, and starch and storage protein biosynthesis-related transcription factor genes were identified as candidate genes for these QTLs (**Supplemental Table 5**). For instance, genes encoding an NAD-dependent epimerase/dehydratase family protein (Teng et al., 2019) and several pentatricopeptide-containing proteins (Hao et al., 2019; Wu et al., 2019) within *DC-2*, *DC-3*, *TP-1*, and *TP-4* may have roles in regulating seed starch synthesis. Within the *DC-3* and *TP-1* intervals, there were several candidate genes potentially associated with carbohydrate metabolism and thus starch synthesis, including genes encoding glycosyl hydrolase, glycosyl transferase, group 1 domain-containing protein, UDP-glucoronosyl and UDP-glucosyl transferase domain-containing protein, and UDP-glucuronate 4-epimerase. A starch synthase (LOC_Os07g22930) within *TP-1* is directly involved in starch synthesis (Sano et al., 1985; Gao et al., 2003; Nakamura et al., 2005; Gao et al., 2011).Two genes encoding the protein transport protein Sec61 within *DC-3* may be involved in storage protein biosynthesis (Wang et al., 2016). Some genes encoding transcription factors such as basic region leucine zipper (bZIP) domain-containing protein and no apical meristem (NAC) protein within the *DC-3*, *CA-3*, and *TP-1* interval were reported to regulate the transcription of starch synthesis enzyme-coding genes (Wang et al., 2013; Zhang et al., 2019).

Previous studies have reported that these kinds of genes regulate seed chalkiness and transparency in rice, corn, and other crops (Wang et al., 2013; Wang et al., 2016; Hao et al., 2019; Teng et al., 2019; Wu et al., 2019; Zhang et al., 2019). For example, the *Flo16* gene encodes an NAD-dependent cytosolic malate dehydrogenase, which plays a vital role in redox homeostasis (Teng et al., 2019). The *flo16* showed the floury endosperm due to reducing the ATP content, resulting in reduced activity of starch synthesis-related enzymes (Teng et al., 2019). The *FLO10* gene encodes a pentatricopeptide repeat protein, which is essential for the trans-splicing of mitochondrial nad1 intron 1 (Wu et al., 2019). A mutation in *FLO10* leads to reduced ATP content and affects starch accumulation in rice, resulting in floury endosperm. The *OsNPPR1* gene encodes a nuclear-localized PPR protein, which affects mitochondrial function (Hao et al., 2019). *osnppr1* mutants show floury endosperm and decreased starch and amylose content. The *GPA4* gene encodes the evolutionarily conserved membrane protein GOT1B, which interacts with rice Sec23, and is involved in mediating the coat protein complex II (COPII) vesicle formation at endoplasmic reticulum exit sites, facilitating anterograde transport of proteins to the Golgi (Wang et al., 2016). *gpa4* mutants accumulate 57 kD glutelin precursors and exhibit defective storage protein biosynthesis, leading to chalkiness. OsbZIP58, a bZIP transcription factor, binds directly to the promoters of six starch-synthesizing genes—*OsAGPL3*, *Wx*, *OsSSIIa*, *SBE1*, *OsBEIIb*, and *ISA2*—to regulate their expression (Wang et al., 2013). *osbizp58* mutants show a white belly in the endosperm. The two endosperm-specific NAC transcription factors, ZmNAC128 and ZmNAC130, in maize bind directly the promoters of the *Bt2* and the *16-kD r-zein* genes to regulate starch synthesis and protein biosynthesis (Zhang et al., 2019). Lack of *ZmNAC128* and *ZmNAC130* caused a shrunken kernel phenotype due to reduced starch and protein synthesis. These findings provided a basis for us to identify certain candidate genes within the QTLs for rice appearance quality traits; however, these candidate genes require further validation.

## Data availability statement

The datasets presented in this study can be found in online repositories. The names of the repository/repositories and accession number(s) can be found in the article/**Supplementary Material**.

## Author contributions

J-PG and X-LC conceived the project and designed the study. S-KJ, L-NX, Q-QY, M-QZ, S-LW, R-AW, TT, L-MH, Q-QG, S-WJ, TS, and Y-JL performed the experiments. S-KJ, L-NX, X-LC, and J-PG analyzed and interpreted the data. S-KJ, L-NX, and J-PG wrote the manuscript. All authors contributed to the article and approved the submitted version.
